# A phase II study of daily carboplatin plus irradiation followed by durvalumab for stage III non-small cell lung cancer patients with PS 2 up to 74 years old and patients with PS 0 or 1 from 75 years: NEJ039A (trial in progress)

**DOI:** 10.1186/s12885-020-07406-y

**Published:** 2020-10-06

**Authors:** Kyoichi Kaira, Atsuto Mouri, Shingo Kato, Kenichi Yoshimura, Hiroshi Kagamu, Kunihiko Kobayashi

**Affiliations:** 1 Department of Respiratory Medicine, Comprehensive Cancer Center, International Medical Center, Saitama Medical University, 1397-1 Yamane, Hidaka-City, Saitama, 350-1298 Japan; 2 Department of Radiation Oncology, Comprehensive Cancer Center, International Medical Center, Saitama Medical University, 1397-1 Yamane, Hidaka-City, Saitama, 350-1298 Japan; 3 Center for Integrated Medical Research, Hiroshima University Hospital, Hiroshima University, 1-2-3 Kasumi, Minami-ku, Hiroshima, 732-8551 Japan

**Keywords:** Durvalumab, Chemoradiotherapy, Locally advanced non-small cell lung cancer, Elderly, PS 2, Carboplatin, Immune checkpoint inhibitor, PD-L1 antibody

## Abstract

**Background:**

Durvalumab is a standard drug used during maintenance therapy after chemoradiotherapy in patients with locally advanced non-small cell lung cancer (LA-NSCLC). However, little is known about the clinical benefits of durvalumab after chemoradiotherapy in patients with LA-NSCLC with a performance status (PS) of 2 and/or aged > 75 years. As daily carboplatin plus concurrent thoracic radiotherapy is recommended for elderly patients according to guideline, the current phase II study aims to investigate the effect of daily carboplatin plus radiotherapy followed by durvalumab for patients with stage III NSCLC who have a PS of 2 and/or are older.

**Methods:**

Daily carboplatin plus radiotherapy followed by durvalumab is performed for the patients with stage III NSCLC who have a PS of 2 and/or are older. This is a trial in progress manuscript.

**Study treatment:**

Daily, intravenous, low-dose carboplatin (30 mg/m^2^ in a 30-min infusion) is administered to patients 1 h before radiotherapy for the first 20 fractions. Radiotherapy for all patients consisted of 60 Gy administered as 30 fractions over 6 weeks. Durvalumab at a dose of 10 mg/kg/body is intravenously administered every 2 weeks for up to 12 months after chemoradiotherapy.

**Exploratory assessment:**

In the future, an exploratory investigation will be performed to determine whether the combined assessment of T-cell markers, PD-L1 expression, and tumor mutation burden could predict the outcomes of the regimen.

**Discussion:**

The results of our study will exhibit the efficacy and tolerability of durvalumab as maintenance therapy after daily carboplatin plus radiotherapy.

**Trial registration:**

During the first registration (before induction chemoradiotherapy), 70 patients will be included; then, we include 58 patients during the second registration (before durvalumab treatment after chemoradiotherapy). https://jcrb.niph.go.jp/.

**Primary endpoint:**

The primary endpoint of the current study is the 12-month progression-free survival (PFS) rate after the initiation of durvalumab.

**Secondary endpoints:**

The secondary endpoints are the feasibility, objective response, PFS, overall survival, and adverse events.

## Background

Chemoradiotherapy is a standard treatment for patients with locally advanced stage III non-small cell lung cancer (NSCLC). Combination regimens used in daily practice include platinum derivatives plus third-generation agents such as cisplatin plus docetaxel [1] and carboplatin plus paclitaxel [2]. However, chemoradiotherapy has not been widely applicable for patients with locally advanced NSCLC (LA-NSCLC). Accordingly, most patients with a performance status (PS) of 0 to 1 and aged less than 70 years receive chemoradiotherapy [1–5]. The remaining patients are treated with radiotherapy alone without chemotherapy and immune checkpoint inhibitors (ICIs). Therefore, the outcomes of these patients are poor, with a 5-year survival rate of < 10% compared to the 5-year survival rate of approximately 15% observed when patients receive chemoradiotherapy [6, 7].

For patients with a poor PS and elderly patients, less toxic therapeutic regimens should be considered in combination with radiotherapy. Carboplatin is a platinum compound that crosslinks with DNA and is associated with a lower incidence of nephrotoxicity, neurotoxicity, nausea, and vomiting than cisplatin [8]. Moreover, carboplatin and cisplatin have similar radiosensitizing properties [9, 10]. A total radiation dose of 60 Gy in 2-Gy fractions with daily carboplatin (30 mg/m^2^ in a 30-min infusion), when administered 1 h before radiotherapy for the first 20 fractions, was found to be effective for prolonging overall survival (OS) with acceptable tolerability [11]. Therefore, per the treatment guidelines in Japan, daily carboplatin plus thoracic concurrent radiotherapy is the recommended treatment.

Carboplatin also has the ability to change the immune status of patients [12]. In fact, part of the antitumor effects of platinum drugs occurs through modulation of the immune system. These immunogenic effects include modulation of STAT signaling; induction of an immunogenic type of cancer cell death through exposure of calreticulin and release of ATP and high-mobility group protein box-1 (HMGB-1); and enhancement of the effector immune response through modulation of programmed death receptor 1-ligand (PD-L1) and mannose-6-phosphate receptor expression. Accordingly, at least part of the antitumor effect of platinum chemotherapeutics may be due to immune-potentiating mechanisms.

Durvalumab is high-affinity, human IgG1 monoclonal antibody that blocks PD-L1 from binding to programmed death 1 (PD-1) and CD80 [13]; a clinical trial proved the antitumor activity of durvalumab in patients with several advanced solid tumors such as NSCLC [14]. The PACIFIC study compared durvalumab to a placebo to determine the prognostic significance of maintenance therapy in patients with stage III NSCLC who received concurrent thoracic radiotherapy with platinum-based chemotherapy [15, 16]. The PACIFIC study demonstrated that progression-free survival (PFS) was significantly longer after durvalumab (16.8 months) than after placebo (5.6 months; stratified hazard ratio for disease progression or death, 0.52; *P* < 0.001). In addition, the 12-month PFS rate was 55.9% (versus 35.3% for placebo). Accordingly, durvalumab after concurrent chemoradiotherapy is widely used for treating patients with locally advanced NSCLC; however, many patients who have a PS of 2 and/or are older are not treated using this combination, because the clinical evidence of this treatment is excluded from the PACIFIC study.

Currently, thoracic radiotherapy alone is the standard of care for elderly patients with LA-NSCLC in Japan, especially those aged > 75 years. However, the combination of daily carboplatin plus concurrent thoracic radiotherapy might be chosen for elderly patients with a good PS and adequate tolerability. On the basis of the evidence of the PACIFIC study, it remains unclear whether a platinum-based regimen plus concurrent thoracic radiation followed by durvalumab results in significant survival prolongation for elderly patients aged > 75 years when compared to chemoradiotherapy with daily carboplatin. Similarly, little is known about the clinical benefit of durvalumab after chemoradiotherapy for LA-NSCLC patients with a PS of 2.

Therefore, the current phase II study aims to investigate the survival benefit of daily carboplatin plus radiotherapy followed by durvalumab for patients with stage III NSCLC, including those who have a PS of 2 and/or are older.

## Methods / design

### Study design and objective

**Fig. 1:**
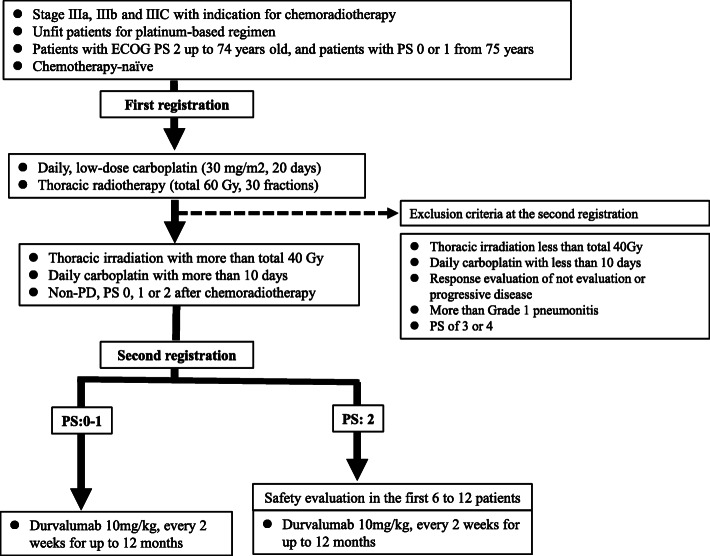
Design and protocol of this study

The current non-randomized, prospective, open-label phase II study aims to evaluate the efficacy of daily carboplatin plus concurrent thoracic radiation followed by durvalumab for patients with stage III NSCLC who have a PS of 2 and/or are older. The design and protocol of this study are shown in Fig. 1.

The primary endpoint of this study is the 12-month PFS rate from the initiation of durvalumab. The secondary endpoints are the feasibility, objective response, PFS, OS, and adverse events. As an exploratory analysis, a predictive biomarker for this regimen will be examined using T-cell markers such as CD62LlowCD4 + T cells and CD25 + Foxp3 + CD4+ in the peripheral blood, and considering the expression of PD-L1 within tumor cells and tumor mutation burden before treatment. All procedures will be performed in accordance with the ethical standards of the institutional and/or national research committee and with the 2013 Declaration of Helsinki and its later amendments or comparable ethical standards. Written informed consent is obtained from all participants in our study. This study has been registered in the Japan Registry of Clinical Trials (JRCT) (jRCTs031190070).

### Key eligibility criteria

The inclusion and exclusion criteria during the first registration are listed in Table 1. For administering durvalumab after chemoradiotherapy with daily carboplatin plus concurrent thoracic irradiation, the second registration will be performed according to the additional inclusion and exclusion criteria, as listed in Table 2.

**Table 1:** Inclusion and exclusion criteria in the first registration

**Inclusion criteria**	
1	Patients with a diagnosis of non-small cell lung cancer (NSCLC) histologically or cytologically
2	Patients that are in stage IIIa, IIIb, or IIIc (UICC 8th version) and have indication for radical radiation therapy, but have no indication for surgical therapy.
3	No previous treatments.
4	Patients with measurable lesions that can be evaluated by RECIST (version1.1).
5	Patients without indication for concurrent chemoradiation using platinum doublet, namely, patients with ECOG PS 2 up to 74 years old, and patients with PS 0 or 1 from 75 years.
6	Normal main organ functions in the following values; Absolute neutrophil count > 1500/mm3, Platelet count ≥100,000/mm3, Hemoglobin ≥9.0 g/dl, AST, ALT ≤2.5 times ULN if no demonstrable liver metastases or ≤ 5 times ULN in the presence of liver metastases, Total bilirubin ≤1.5 times ULN if no liver metastases or ≤ 3 times ULN in the presence of documented Gilbert’s Syndrome [unconjugated hyperbilirubinaemia] or liver metastases, Creatinine within the normal value in each institution, PaO2 or SpO2 ≥ 70 Torr or ≥ 95%
7	Written informed consent must be given
Exclusion criteria	
1	Past medical history of interstitial lung disease, drug-induced interstitial lung disease, or any evidence of clinically active interstitial lung disease
2	Contralateral hilar nodes, atelectasis of the entire lung, or malignant pleural or pericardial effusions
3	Patients with severe complications such as uncontrolled heart, lung, liver, or kidney disease or diabetes mellitus
4	Any of the following cardiac criteria: Patients must not enter the study if any of the following exclusion criteria are fulfilled: i) Mean resting corrected QT interval (QTc) > 470 msec obtained from 3 electrocardiograms (ECGs), using the screening clinic ECG machine derived QTc value, ii) Any clinically important abnormalities in rhythm, conduction or morphology of resting ECG e.g. complete left bundle branch block, third degree heart block and second degree heart block. iii) Any factors that increase the risk of QTc prolongation or risk of arrhythmic events such as heart failure, hypokalaemia, congenital long QT syndrome, family history of long QT syndrome or unexplained sudden death under 40 years of age in first degree relatives or any concomitant medication known to prolong the QT interval.
5	Any evidence of active infection including hepatitis B, hepatitis C and human immunodeficiency virus (HIV)
6	Patients with severe malabsorption syndrome or with disease affecting digestive function such as total gastrectomy or active inflammatory bowel disease
7	Patients that have received systemic administration of steroids at a dose of more than prednisolone10mg (or equivalent to this) for 4 weeks or longer
8	Besides the above-mentioned cases, those with contraindications for therapy with carboplatin
9	Patients with active double cancers other than intramucosal carcinoma
10	Mixed small cell and non-small cell lung cancer histology
11	Any concurrent chemotherapy, immunotherapy, biologic or hormonal therapy for cancer treatment. NOTE: Local treatment of isolated lesions, excluding target lesions, for palliative intent is acceptable (eg, by local surgery or radiotherapy)
12	Active or prior documented autoimmune disease within the past 2 years. NOTE: Patients with vitiligo, Grave ‘s disease, or psoriasis not requiring systemic treatment (within the past 2 years) are not excluded.
13	Active or prior documented inflammatory bowel disease (eg, Crohn ‘s disease, ulcerative colitis)
14	History of primary immunodeficiency
15	History of organ transplant that requires therapeutic immunosuppression
16	History of another primary malignancy within 5 years, except for adequately treated basal or squamous cell carcinoma of the skin or cancer of the cervix in situ and the disease under study
17	Other cases that are determined to be inappropriate by attending physicians

**Table 2:** Additional inclusion and exclusion criteria in the second registration

Inclusion criteria	
1	Completion of thoracic irradiation with more than total 40 Gy
2	Daily carboplatin with more than 10 day
3	Non-PD and PS 0, 1 or 2 after chemoradiotherapy
4	Normal main organ functions in the following values; Absolute neutrophil count > 1500/mm3, Platelet count ≥100,000/mm3, Hemoglobin ≥9.0 g/dl, AST, ALT ≤2.5 times ULN if no demonstrable liver metastases or ≤ 5 times ULN in the presence of liver metastases, Total bilirubin ≤1.5 times ULN if no liver metastases or ≤ 3 times ULN in the presence of documented Gilbert’s Syndrome [unconjugated hyperbilirubinaemia] or liver metastases, Creatinine within the normal value in each institution, PaO2 or SpO2 ≥ 70 Torr or ≥ 95%
Exclusion criteria	
1	Patients with locally advanced NSCLC who have progressed whilst radiotherapy concurrent with daily carboplatin
2	Any unresolved toxicity CTCAE >Grade 2 from the prior chemoradiation therapy
3	Patients with any grade pneumonitis from prior chemoradiation therapy
4	Uncontrolled intercurrent illness including, but not limited to, ongoing or active infection, symptomatic congestive heart failure, uncontrolled hypertension, unstable angina pectoris, cardiac arrhythmia, active peptic ulcer disease or gastritis, active bleeding diatheses including any patient known to have hepatitis B, hepatitis C or human immunodeficiency virus (HIV), or psychiatric illness/social situations that would limit compliance with study requirements or compromise the ability of the patient to give written informed consent
5	Receipt of live attenuated vaccination within 30 days prior to study entry or within 30 days of receiving Durvalumab
6	Recent major surgery within 4 weeks prior to entry into the study (excluding the placement of vascular access) that would prevent administration of Durvalumab.

Abbreviation: *PD* progressive disease; *PS* performance status; AST,; ALT,; NSCLC, non-small cell lung cancer; CTCAE,

### Treatment schedule

Daily, intravenous, low-dose carboplatin (30 mg/m^2^ in a 30-min infusion) is administered 1 h before radiotherapy for the first 20 fractions. Planned radiotherapy of 60 Gy is administered as 30 fractions from 6 to 9 weeks. Radiotherapy is administered 5 days per week (i.e., Monday to Friday with the weekend off) in 2 Gy daily fractions via 6–18 MV X-rays. Both three-dimensional conformal radiotherapy and intensity-modulated radiotherapy are acceptable. Radiation doses are prescribed according to the planning target volume. Motion management is performed as required, and the internal target volume, clinical target volume, and planning target volume vary according to the motion management method used. The use of positron emission tomography (PET) or computed tomography (CT) and four-dimensional CT for radiotherapy planning is encouraged. The gross tumor volume is defined as the primary tumor and any regionally involved nodes on CT (> 1 cm on the short axis) or pretreatment PET (standardized uptake value > 3). The internal target volume is defined as the envelope that encompassed the gross tumor volume plus the ventilatory motion. The clinical target volume margins are 0·5–1·0 cm beyond the internal target volume. The planning target volume margins are 0·5–1·5 cm beyond the clinical target volume, depending on the use of four-dimensional CT for planning and image-guided radiotherapy for delivery. The V20 (the volume of the lung parenchyma that received 20 Gy or more) needs to be less than 30%.

Durvalumab is administered 1 to 42 days after the patients receive chemoradiotherapy. Durvalumab is intravenously administered at a dose of 10 mg per kilogram of body weight every 2 weeks for up to 12 months.

### Statistical analysis

The primary endpoint is to evaluate the progression free survival rate of 12 months from the initiation of durvalumab administration. The secondary endpoint is to investigate the time to failure, the rate of therapeutic completion, progression-free survival, 2-year survival rate, objective response rate, safety and exploratory biomarker assessment. In the PACIFIC study, the 12-month PFS rate was 55.6% after chemoradiotherapy followed by durvalumab and 35.3% after chemoradiotherapy alone. In the JCOG0301 study, the 12-month PFS rate was approximately 30% after radiotherapy with daily carboplatin [11]. According to the retrospective study conducted by Arslan et al. [17], the 1-year OS rate of patients with LA-NSCLC with a PS of 2 was approximately 50%. This was lower than the 1-year OS rate for elderly patients with a good PS (70.8%) observed in the JCOG0301 study. The degree of difference in OS between patients with a poor PS (included in the study by Arslan et al.) and elderly patients (included in the JCOG0301 study) is approximately 30%, which could be applied to the difference in PFS between these populations. Therefore, we assumed that a 12-month PFS rate of 35% after radiotherapy with daily carboplatin followed by durvalumab in eligible patients would indicate potential clinical usefulness, whereas a rate of 20% would be the lower limit for indicating clinical usefulness. On the basis of this assumption, our study was designed to have a power of 80% and had an alpha level of 0.1 (two-sided). The target number of patients for this study is 53. Therefore, we assess 58 patients considering the progressive disease (PD) rate after treatment with radiation with daily carboplatin and patients who dropped out. In the JCOG0301 study, the PD rate was 8%, the rate of treatment-related death was 3%, and the rate of Grade 3–4 adverse events was 6%, so we assumed the dropout rate to be 20%. During the first registration, 70 patients will be included; then, we analyze 58 patients included during the second registration. A minimum follow-up of 12 months is needed after the last patient starts treatment with durvalumab.

Safety evaluation:

We are also investigating the safety in the first 6 patients with a PS of 2.

**Fig. 2:**
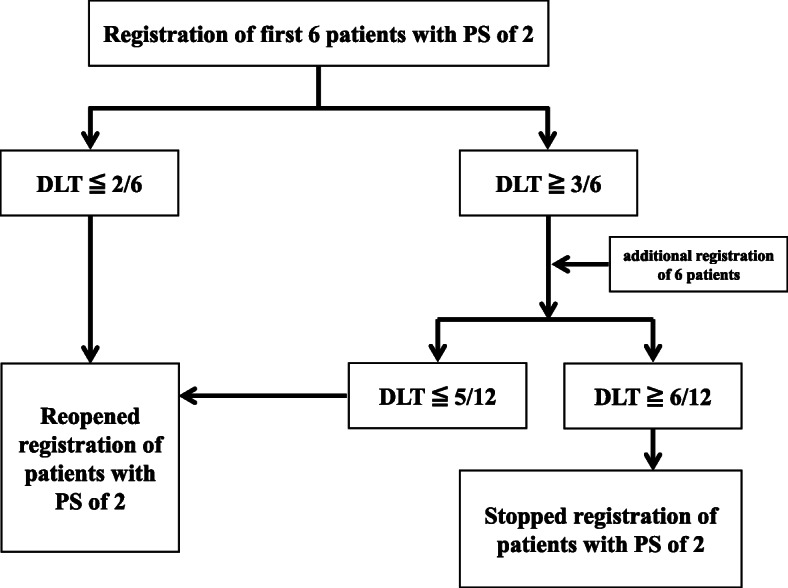
Safety evaluation in 6 to 12 patients with PS of 2

Further enrollment of patients with a PS of 2 will be stopped until the safety is confirmed in these 6 patients according to the following criteria (Fig. 2):

• Patients will be considered to have dose limiting toxicities (DLTs) if durvalumab treatment is interrupted more than two times owing to >Grade 2 pneumonitis or the occurrence of >Grade 3 adverse events.

• The DLTs will be evaluated during the period of 8 weeks after the administration of durvalumab 4 times.

• If < 2 patients show DLTs, durvalumab will be deemed to be safe, and the inclusion of more patients with a PS of 2 will be restarted.

• If 3 DLTs are observed in the first 6 patients, 6 more patients will be included; then, if fewer than 5 of the 12 patients experience a DLT, the enrollment of patients with a PS of 2 will be restarted. Of the 12 patients, if 6 or more experience a DLT, further enrollment of patients with a PS of 2 will be stopped.

### ParticipatXing institutions

The participating institutions include 31 hospitals in Japan.

## Discussion

This is the first prospective study to evaluate the efficacy and feasibility of durvalumab as maintenance therapy after chemoradiotherapy for patients with unresectable stage III NSCLC who have a PS of 2 and/or are older (i.e., aged > 75 years). In particular, it remains unclear whether durvalumab could display a significant clinical benefit in patients with a PS of 2 after chemoradiotherapy; therefore, the results of our study are expected to elucidate this clinical difficulty. The JCOG0301 study demonstrated that the 5-year survival rate was approximately 15%, which was less than 20%, observed in elderly patients with LA-NSCLC who received daily carboplatin plus concurrent radiotherapy [11]. In future, we expect that the 5-year survival rate will increase to > 20–30% after additional treatment with durvalumab, although our investigation is a phase II study to evaluate the 12-month PFS rate from the initiation of durvalumab. Till date, no prospective study has elucidated the clinical significance of chemoradiotherapy for patients with LA-NSCLC with a PS of 2. Although anti-PD-1/PD-L1 antibodies result in a significant survival benefit for patients with advanced NSCLC with a good PS [18], the efficacy of ICIs is known to increase after radiotherapy [19]; therefore, a synergistic effect is expected for patients with a PS of 2. If mild toxic regimens, such as daily carboplatin—as used in the current study—are combined with concurrent thoracic radiotherapy, the regimen might be feasible for patients with a PS of 2, and sequential therapy with durvalumab will have the potential of improving the survival of such patients. Similarly, ICIs are feasible and effective for elderly patients with NSCLC (aged ≥ 75 years) [18], and we believe that durvalumab as maintenance therapy after chemoradiotherapy is suitable for such patients.

As biomarkers, PD-L1 and the tumor mutation burden are broadly useful for predicting the efficacy of ICIs in patients with advanced NSCLC [19–21]. Recently, we reported that the increased level of CD62LlowCD4 + T cells in the peripheral blood could effectively predict the efficacy and survival of patients with advanced NSCLC who received anti-PD-1 antibodies [22]. It remains unclear whether the value of CD62LlowCD4 + T cells is closely associated with the survival benefit of durvalumab after chemoradiotherapy; however, our exploratory investigation is necessary, as the results will help in the selection of patients who are sensitive to durvalumab.

## Conclusion

The results of the NEJ039A study will exhibit the efficacy and tolerability of durvalumab as maintenance therapy after daily carboplatin plus radiotherapy, which is among the potential promising first-line treatment regimens for patients who have a PS of 2 and/or are older (aged > 75 years). Moreover, the results of our exploratory investigation are expected to confirm whether the combined assessment of T-cell markers, PD-L1 expression, and tumor mutation burden could predict the outcomes of this regimen.

## Data Availability

Not applicable.

## Abbreviations

LA-NSCLC: Locally advanced non-small cell lung cancer
PS: Performance status
PFS: Progression-free survival
NSCLC: Non-small cell lung cancer
HMGB-1: Overall survival: OS; high-mobility group protein box-1
PD-L1: Programmed death receptor 1-ligand
PD-1: Programmed death 1
JRCT: Japan Registry of Clinical Trials
PET: Positron emission tomography
CT: Computed tomography
DLTs: Dose limiting toxicities
